# Psychotropics in different causes of itch: systematic review with controlled studies*

**DOI:** 10.1590/abd1806-4841.20164878

**Published:** 2016

**Authors:** Lízie Emanuelle Eulalio Brasileiro, Dayanna Patrícia de Carvalho Barreto, Emerson Arcoverde Nunes

**Affiliations:** 1 Universidade Federal do Rio Grande do Norte (UFRN) – Natal (RN), Brazil; 2 Universidade de São Paulo (USP) – São Paulo (SP), Brazil

**Keywords:** Pruritus, Psychotropic drugs, Controlled clinical trial

## Abstract

Among the wide range of symptoms neglected or resistant to conventional treatments in clinical practice, itch is emerging gradually as a theme to be studied. Itch complaints and the negative effects in the quality of life are observed in several medical fields. Although the partially obscure pathophysiology, some researchers decided to check and test the use of psychotropic drugs in resistant itch to conventional topical treatments and antihistamines. The objective of this study was to evaluate scientific evidence in psychotropic use in the treatment of itch of various causes. This is a systematic review of scientific literature. The following databases were used: PubMed, Web of Science, Scopus and Scielo. Randomized controlled trials that should focus on treatment with psychotropic drugs of pruritus of various causes were the inclusion criteria. All articles were analyzed by the authors, and the consensus was reached in cases of disagreement. Fifteen articles were included after analysis and selection in databases, with the majority of clinical trials focusing on psychopharmacological treatment of itch on account of chronic kidney disease. Clinical trials with psychotropic drugs mostly indicated significant improvement in the itching. In most trials of chronic kidney disease as basal disease for itch, greater effectiveness was observed with the use of psychotropic drugs compared with placebo or other antipruritic. However, the small amount of controlled trials conducted precludes the generalization that psychiatric drugs are effective for itch of various causes.

## INTRODUCTION

Itch was defined for a long time as an unpleasant cutaneous sensation that provokes the desire to scratch.^1^ The chronicity of itch involves multidimensional phenomenon that includes physiological (as the central neurons and nerve pathways), cognitive and emotional aspects.^1,2^ Itch can be classified into four dimensional categories, according to the neurophysiologic considerations: pruritoceptive, with peripheral origin; neuropathic; neurogenic; and psychogenic, with central origin.^3-5^ The diagnosis of psychogenic itch has the need to discard skin causes or underlying systemic disease, representing a diagnostic challenge.^6,7^

When the itch is related to chronic diseases or diseases that cause major limitation for life, such as chronic kidney disease and liver or skin diseases, it can cause considerable influence on the behavior and, just like pain, it can cause loss of quality of life. ^8-10^

The itch sensation originates in free nerve endings in the skin and is transmitted by C fibers to the dorsal horn of the spinal cord and then to the cerebral cortex via the spinothalamic tract.^11,12^ There are no itch specialized receptors at the end of the peripheral nerves, but the specificity of the neurons that transmit the itch is in connection with the spinal cord tracts.^9^

As a consequence of chronic itch, a person may have skin lesions caused by excessive scratching, pinching (ulcers, secondary infections, bleeding, permanent discoloration or scarring), just as it can cause sleep disturbance, depression, overall stress or anxiety, among other psychiatric disorders.^9,10,13^

The treatment of itch often include antihistamines, antidepressants, opioid antagonists and 5HT_3_ receptors, since endogenous pruritogenic mediators (serotonin, histamine, neuropeptides, prostaglandins, endogenous opioids) have been implicated in triggering pruritic stimulus.^15,8,14^ Often, chronic itch is refractory to conventional therapies, which leads to the search for medications initially underused as psychotropics, reason why clinical trials are being conducted in this area.

It is worth mentioning that pharmacological therapy that has been used in clinical practice is based on small studies, and that there are few controlled clinical trials.

This paper is a systematic review, which aims to observe the published studies that demonstrate the effectiveness of psychotropic drugs in different primary causes of itch.

## MATERIAL AND METHODS

This review was conducted following PRISMA (*Preferred Reporting Items for Systematic Reviews and Meta-Analysis*) guideline.

The preparation of the systematic review was initiated in December 2014, initially through retrospective consultation of electronic databases *PubMed, Web Of Science, Scopus and Scielo,* using the following keywords: *Pruritus, itch, treatment, psychotropic drugs*, organized as follows: "*pruritus" OR "itch" AND treatment AND psychotropic drugs,* and their descriptors in Portuguese for searching in *Scielo*.

Included articles should be Controlled Trials, in humans, in English or Portuguese. Articles focusing on drugs other than psychotropic drugs or on diseases that didn't have itch as a studied variable were excluded from the analysis.

The first step consisted of reading the titles and abstracts, and selecting articles that contained the keywords previously explained or derived words (MeSH terms). Based on selected articles, the second step consisted of thorough and independent analysis of each article by the authors, assessing the consistency with the proposed theme and methodological rigor. The authors met before the collection to ensure uniform understanding of the eligibility criteria. In cases of disagreement, the authors tried to reach a consensus.

Finally, in order to find useful items that were not contained in the initial search, the reference lists of articles originally included were used.

## RESULTS

**Figure 1:**
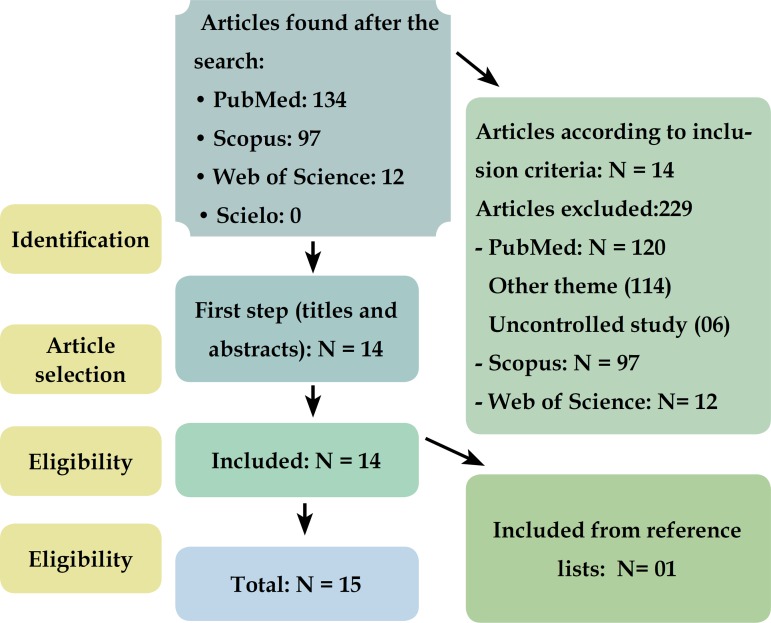
Flowchart. Process of selection of articles

Based on the terms used for the search and specifiers, 134 articles were found in PubMed; 12 articles in Web of Science; 97 in Scopus; and 0 in Scielo database. All items found were in English. Among these, 15 articles fulfilling the inclusion criteria were included in this systematic review. From PubMed database, 114 articles were excluded because they were not related to the keywords and 06 for not being controlled clinical trials; 01 article was included from a reference list (Figure 1). In the other databases, all items were excluded because none was a controlled trial.

We observed significant heterogeneity in clinical trials as the primary types that led to itch as well as to the psychotropic and methodologies used.

The articles included in this review presented as the origin of itch: chronic kidney disease or dialysis (6); itch after burn (2); mustard gas lesions and itch for other dermatological causes (2); chronic liver disease or cholestasis (2); chronic idiopathic urticaria (2); and one for not dermatologic causes (with a predominance of cancer). It should also be evidenced that there has been no controlled study addressing the psychogenic or somatoform as an origin of itch.

The psychotropic drugs used in these clinical trials for the various causes of itch were: pregabalin, gabapentin, doxepin, paroxetine and sertraline. It was decided that clinical trials comprised in this review would be presented grouped by the cause of itch to allow some inferences on the observed heterogeneity of etiologies. Where it was not feasible a more homogeneous grouping, we performed the methodological, results and other possible outcomes descriptions of each study.

### Itch secondary to chronic kidney disease or dialysis

Table 1 shows the six clinical trials included in the study on itch due to chronic kidney disease.

**Table 1:** Itch secondary to chronic kidney disease (CKD)

Study	Country	Design	N of subjects	Drugs	Via	Duration	Instrument	Type of itch	Results
Solak Y et al., 2012	England	Prospective, randomized, crossover, comparative	50	Gabapentin 300 mg 3x/ week Pregabalin 75mg/day	Oral	14 weeks (6 weeks for each drug; 2 weeks WO)	VAS	Peripheral neuropathy in patients in dialysis	Significantly decrease in symptoms, with no difference between the two drugs
Marquez D et al., 2012	Argentina	prospective, nonrandomized,	22	Gabapentin 300 mg 3x/ week of desloratadine 5mg 3x/week	Oral	7 weeks (3 weeks each drug; 1 week WO)	VAS	dialysis	Only desloratadine had statistical significance
Gunal AI et al., 2004	Turkey	Prospective, randomized, placebo-controlled, double-blind	25	Gabapentin 300mg or placebo	Oral	9 weeks (4 weeks each drug; 1 week WO)	VAS	dialysis	Decreased score showed highly significance (0.0001) after gabapentin
Razeghi E et al., 2009	Iran	Prospective, placebo-controlled, double-blind	34	Gabapentin 100mg or placebo 3x/weeks	Oral	9 weeks	VAS	dialysis	Effectiveness of gabapentin in low doses
Naini AE et al., 2007	Iran	Longitudinal, randomized placebo-controlled	34	Gabapentin 400mg Placebo	Oral	4 weeks	VAS	dialysis	Gabapentin was effective
Pour- Reza- GholiF. et al., 2007	Iran	Prospective, randomized, placebo-controlled, double-blind, crossover	24	Doxepin 10mg 2x/day Placebo	Oral	3 weeks (1 week each group; 1 week WO)	Clinical improvement	dialysis	Doxepin with significant improvement

Legend: WO- washout; VAS: Visual Analogue Scale

In the six studies of patients with chronic kidney disease (CKD) at advanced stage, and therefore performing hemodialysis (HD), there was the participation of a total of 179 individuals with a mean age of 56 years (± 11.8).

In most of the six studies, the characteristics of patients was similar as well as the criteria that led to the selection of the participating subjects. As inclusion criteria, the subjects should be at least 3 to 6 months in hemodialysis, with a frequency of 2 to 3 sessions per week, with stabilized patterns (normal calcium, phosphatase, parathyroid hormone, aluminum, serum magnesium and hemoglobin values). They should also present medium to severe intensity itch, with duration varying from 6 to 8 weeks, not respondent to traditional pre-treatments (antihistamines and emollients). Exclusion criteria followed the same line, therefore subjects with liver, dermatologic and other metabolic disorders were excluded from the study. Solak *et al.* stablished broader criteria, thus excluding individuals with uncontrolled mental illness.^15^ The antipruritic agents used should be suspended at least 1 week before the start of the intervention.

Itch, just like pain, is a subjective and difficult to quantify symptom, given its subjectivity. Thus, among the six clinical trials, five used the Visual Analogue Scale (VAS) for measuring the symptomatic itching before, during and after surgery. Therefore, the results assumed are based on the changes observed over this scale. Only in the trial conducted by Pour-Reza-Gholi *et al.* a different method was used, through the categorization of subjective improvement: complete improvement, relative improvement and no effect, respectively.^16^

We found that, of the six trials on CKD as underlying disease for itch, five used gabapentin 100-300 mg following dialysis sessions as medication to be compared and/or tested. Doxepin was used in one study on the grounds that, until then, it had not been tested in patients with CKD in controlled trials.^16^ The rationale for the use of such tricyclic antidepressant would be because it presents strong anti-histaminergic action, acting in one of the neurotransmitters of itch mechanism.^16^

Among these six studies, we observed that in five of them there was a significant improvement of itch with use of psychotropic, although Solak *et al.* study compared gabapentin 300 mg, 3 times a week, with pregabalin 75 mg daily, two anticonvulsants.^15^ Interestingly, in one of the trials, the outcome was different. Marquez *et al.* compared a group using desloratadine 5 mg/day (3 weeks) with other group using gabapentin 300 mg after hemodialysis sessions (3 weeks), with cross over and a week washout. The study showed that desloratadine presented significant improvement in itch, while gabapentin, although it also improved the symptom, was not statistically significant.^17^ Furthermore, three of the six studies presented very similar methodologies, but one of the differences observed in these studies was the dosage of gabapentin 300 mg 3 times a week, 400 mg 2 times a week and 100 mg 3 times a week.^18-20^ These three trials demonstrate the effectiveness of gabapentin over the placebo group.^18-20^

As for secondary endpoints, all six trials demonstrated greater adverse events in the groups using psychotropics, especially dizziness, drowsiness and fatigue, more intense after the first dose and with gradual tolerance. However, some subjects could not continue in the trial due to the intensity and persistence of adverse events. This occurred in the study of Marquez *et al.*, in which four subjects discontinued the study due to gabapentin (fatigue and drowsiness) and one abandoned it when used desloratadine due to "nervousness". ^17^ In Razeghi *et al.*, there was a loss caused by adverse events of gabapentin. ^20^

### Itch with dermatological cause

Two articles included in this review addressed different skin impairment (in sequence, lesion resulting from sulfuric gas and atopic eczema), so it was not possible to unify methods or actions to be taken, although the tested medication was the same, doxepin cream 5% (Table 2).

**Table 2:** Itch secondary to skin diseases

Study	Country	Design	N of subjects	Drugs	Via	Duration	Instrument	Type of itch	Results
Greene et al., 1985	USA	Longitudinal, controlled, double-blind, randomized	50	Doxepin 10mg or diphenhydramine 25mg 3x/day	Oral	31 days	McNemar test	Chronic idiopathic urticaria	Itch decreased with doxepin according to the instrument used
Goldsobel et al., 1986	USA	Longitudinal, placebo-controlled, double-blind, randomized	16	Doxepin 25mg Placebo 3x/day	Oral	8 weeks	Clinical evaluation	Chronic idiopathic urticaria	Itch decrease with doxepin
Panahi Y et al., 2011	Iran	Prospective, randomized, single-blind	75	Doxepin cream 5% or betamethasone cream	Topic	6 weeks	VAS Itch score DLQI	Lesion due to mustard gas	Significant decrease of pruritus with both topical drugs
Groene D et al., 2001	Germany	Longitudinal, placebo-control, randomized, crossover	11	Doxepin cream 5%; Wolff basis cream (placebo)	Topic	4 days	SDS (self-rating depression scale)	Atopic eczema	No significant antipruritic action of doxepin compared with basis cream

Legend: WO- washout; VAS: Visual Analogue Scale

Panahi's clinical trial aimed to check whether doxepin could be an effective alternative to relieve the itching caused by mustard gas. The study included 75 men who were exposed to mustard gas decades ago and who presented itch. Subjects were excluded if they presented itch secondary to systemic or cutaneous disease, not triggered by mustard gas. Patients were divided into two groups: in one group (40 men), doxepin 5% was applied in lesions two times a day; in the other group (35 males), betamethasone 0.1% was applied for 6 weeks. ^21^

It was found a significant improvement in almost all affected parts of the body in both groups, with no significant difference between groups, according to VAS and DLQI (Dermatology Life Quality Index). Doxepin cream 5% resulted in fewer adverse events compared with betamethasone topical. Topical presentation of doxepin compared with oral presentation presented advantages because it did not cause adverse events typical of tricyclic antidepressants. ^21^

Groene *et al.* conducted a quite diverse clinical trial in terms of methodology compared with the studies above. He performed the induction of itch by acetylcholine in order to test doxepin cream 5% on itch improvement in patients with atopic eczema. The study used the front side of the forearms of 11 subjects, randomizing them, with the aim of comparing doxepin cream 5%, applied to a forearm, with placebo, applied to the other forearm, 4 times a day during 3 days. In the following day (Day 4), acetylcholine or sodium hydrochloride applications were made in the pretreated regions. It was observed that doxepin didn't have a greater antipruritic effect when compared with placebo, and the author referred it should be due to the moisturizing effect of the base solution used as a placebo, although the dimension of the papule was lower in the forearm that received doxepin, suggesting that acetylcholine caused less reaction. ^22^

### Itch for chronic idiopathic urticaria

Also in dermatologic causes, in the 1980's two studies were performed with doxepin oral for the treatment of chronic idiopathic urticaria, with similar methodology, not replicated until the time of writing this article (Table 2).

In 1985, Greene *et al.* published a study of 14 months duration in which 50 patients were evaluated in a randomized double-blind controlled trial of diphenhydramine 75 mg/day.^23^ The study included patients refractory to previous treatments and excluded patients younger than 18 years old, pregnant and nursing women. The sample was divided into three groups of types of urticaria as follows: simple urticaria, leukocytoclastic urticaria and lymphoblastic urticaria; and it was also subdivided by severity.

This trial showed clinical and statistical superiority of doxepin at a dosage of 30 mg/day, with complete remission of symptoms in 43% of patients compared with 5% remission when in use of diphenhydramine (p<0.001). The study also showed 74% of partial improvement in symptoms with the use of antidepressant, compared with 10% improvement with the use of antihistamine (p<0.001), being, therefore, a safe alternative for the treatment of patients with simple itch that do not respond to common antihistamines. Despite these positive results, the only statistical test used was the McNemar test, which is used to compare dichotomous variables, which was not the case of this study that quantified the symptoms in four categories. Thus, the inadequate statistical analysis limited the conclusions presented by the authors.^24^

In the following year, Goldsobel *et al.* also published a double-blind controlled study, this time with placebo, with duration of four weeks.^25^ This study included a sample population of 16 patients, 14 women and 2 men, but not all had undergone previous treatment (1 patient). This trial also showed clinically and statistically significant results of doxepin, with improvement in all evaluated categories, when compared with placebo: 51% reduction in number of lesions (2.02 versus 0.98 placebo; p<0.001), 64% reduction in discomfort with the symptoms (1:38 versus 0.49 placebo; p<0.001), and 75% reduction in edema (0.85 versus 0.21 placebo; p<0.001).

Despite this improvement, methodological flaws limited incisively the conclusions from these observations. For example, the symptoms were grouped into categories, resulting in ordinal variables, which were compared using paired t-tests, which are applied to mean of interval variables, beacuse it does not make sense to calculate mean of ordinal of categorical variables. Thus, the data presented as statistically significant actually were not calculated properly. ^24^

### Itch for chronic liver disease (CLD) or cholestasis

Two clinical trials were conducted in 2006, randomized, placebo-controlled, totaling 28 subjects with chronic liver disease (the leading cause of both studies was the primary biliary cirrhosis) and presence of itch (Table 3). The factors necessary to enter in both studies were: evidence of cholestatic liver disease and itch over three months. The exclusion criteria in common were: patients could not have dermatologic disease associated with itch or make use of antipruritic agents. These, if used, should be suspended until two weeks before starting the study; also, patients could not be taking antidepressant, opioid, corticosteroids, phenothiazines and antiviral for hepatitis or they should be suspended five days before the start of the trial.^26,27^ Bergasa et al. proposed further exclusion criteria: history of hepatic encephalopathy, ascites, visceral bleeding, malignancy, cannot prevent pregnancy or being pregnant, creatinine greater than 1.7 mg/dL, hemoglobin less than 10 g/dL, have received a liver transplant or having human immunodeficiency virus.^27^

**Table 3:** Itch secondary to chronic liver disease; malignancies; after-burns

Study	Country	Design	N of subjects	Drugs	Via	Duration	Instrument	Type of itch	Results
Ahuja RB et al., 2012	India	Randomized, double-blind, placebo-controlled	80	G1: cetirizine and chlorpheniramine; G2: pregabalin, cetirizine and chlorpheniramine; G3: Vit.B G4: Pregabalin	Oral	9 months	VAS	After burns	Decrease in symptoms significantly, with no difference between the two drugs
Ahuja RB et al., 2010	India	Longitudinal, randomized, double-blind	60	G1: Cetirizine 10mg G2: Gabapentin 300mg G3: Gabapentin + cetirizine	Oral	4 months	VAS	After burns	VAS reduced 95% in the gabapentin group (52% in the cetirizine)
Mayo MJ et al., 2006	USA	Randomized, placebo- controlled, double-blind	12	Sertraline 75-100mg per day, and placebo	Oral	13 weeks	VAS	CLD	Sertraline shown to be statistically significant
Bergasa NV et al., 2006	USA	Randomized, placebo-controlled, double-blind	16	Gabapentin 100 mg to 2400mg / day or placebo	Oral	4 weeks	VAS HSA HDRS SCID	CLD	Gabapentin demonstrated no significant improvement in itch
Zylicz Z et al., 2003	Netherlands	Prospective, randomized, placebo-controlled, double-blind, crossover	26	Paroxetine 20 mg or placebo	Oral	Variable according to the response of each subject	NAS (Numerical Analogue Scale)	No dermatological (malignancy)	Effectiveness of paroxetine

Legend: SCID: Structured Clinical Interview; DLQI: Dermatology Life Quality Index); HDRS: Hamilton Rating Scale for Depression; CLD: chronic liver disease

Both studies were randomized, placebo-controlled and double-blind. However, both compared different medications with placebo, in view of possible different pathophysiological mechanisms of pruritus. Mayo *et al.* used sertraline, a serotonin receptor inhibitor, understanding that the serotonergic pathway is important in the perception of itch.^26^ Bergasa *et al.*, differently, used gabapentin, having the hypothesis that the itch is part of nociceptive stimulation and therefore would improve with this medication.^27^

Mayo *et al.* study was composed of two phases: the first had the function of dose titration of sertraline in 38 subjects, as well as laboratory monitoring of sertraline and its metabolite; the second, after excluding 26 subjects, had the function of demonstrating the effectiveness of sertraline. In this second phase, VAS and evaluation of lesions secondary to itch were used during follow-up for 13 weeks, and a higher effectiveness was verified, with significance in the group using sertraline 75 mg to 100 mg daily compared with placebo.^26^

It was also observed that doses higher than 100 mg/day does not necessarily caused benefits related to itch, but increased adverse events like insomnia, fatigue and increased bowel habits.

In the clinical trial conducted by Bergasa *et al.*, it was found unexpected and initially paradoxical result. It was concluded that there was no significant improvement after therapeutic trial of four weeks in the group receiving gabapentin in variable dose of 300 mg to 2400 mg per day, compared with placebo, in aspects related to the assessment of itch (little decrease in VAS) and frequency in scratching (HSA-Hourly Scratching Activity). In apparent contradiction, it was observed that the HSA of patients in the placebo group decreased and approached zero, which allowed the conclusion that there was a subjective improvement of itch and a change in behavior. ^27^

Also the adverse events observed in the group receiving gabapentin were: increase in serum bilirubin (1 patient), fatigue, dizziness, worsening of symptoms of carpal tunnel syndrome, vomiting, and fluctuations in serum creatinine.^27^ Another outcome observed in this trial was the presence of depressive symptoms – assessed by HDRS (Hamilton Depression Rating Scale) – presented mildly in eight patients, and moderate in three and absent or minimal in two patients; and by SCID (Structured Clinical Interview Questionnaire) axis 1 – 12 patients were classified with mood changes due to general medical condition and one with depressive episode with atypical presentation.

### Itch after burning

Two articles related to burning as a primary cause of itch were included, both with methodological design of randomized, double-blind, and only the first described below held control with placebo group (Table 3).

Inclusion criteria were nearly identical between the two clinical trials as well as the exclusion criteria. To enter these studies, patients should be aged between 18-60 years (in the second study, between 12-70 years), present percentage of burned body area higher than 5%, including predominance of second degree burns and lesions in healing process or already healed up to 3 months. As exclusion criteria, the same comorbid conditions were marked: diabetes mellitus, skin disease, kidney disease, pregnancy, lactation or surface of grafted area greater than 2%.

In the first study, Ahuja conducted a clinical trial with 80 subjects who were subdivided into four groups, divided equally, arranged in the subsequent order: cetirizine and pheniramine maleate (antihistamine group); pregabalin, cetirizine and pheniramine maleate (combined group); placebo (vitamin B complex); and another group which received only pregabalin. In this trial, VAS was used not only for subsequent itch ratings, but also to determine the dosage of each drug used in accordance with the categorization in mild itch (2-5 VAS), moderate itch (6-8) and severe itch (9 and 10). Therefore, cetirizine doses ranged from 10 to 30 mg/day; pregabalin doses ranged from 75 to 300 mg; and pheniramine doses was maintained at 25 mg/da.

In mild to moderate itch, the almost complete remission was achieved in the combined and pregabalin group, with a significant difference compared with the antihistamine and the placebo group. Regarding the severe itch, there was also a significant improvement in the combined and pregabalin groups when VAS was evaluated on the 28th day. There were no reported adverse events in the placebo and pregabalin groups, however, the antihistamine and combined groups, 11 and 9 patients complained of drowsiness, respectively.^28^

The second trial included in this review was conducted by the same author, with the objective to test gabapentin in itch secondary to severe burns as well as to test if the association of gabapentin to an antihistamine would result in significant superiority of the junction of the two neurophysiological mechanisms.^29^ Therefore, 60 patients were divided into three groups with dosage quantitatively related to VAS: A-Cetirizine 10 to 20 mg/day; B - Gabapentin 300 to 900 mg/day; C-Cetirizine and gabapentin. The study concluded that there was significant difference in the decrease of itch assessed by VAS when comparing group A (52%) with group B (95%). Also, all patients in group B presented complete improvement in itch on the 28th day, compared with 3 patients (out of 20) in group A. There was no significant difference between groups B and C. ^29^

### Itch for non-dermatological causes (malignant diseases)

A single study was included, whose central theme was the psychopharmacological treatment of non-dermatological itch due to, mostly, malignancies and serious systemic diseases in order to test paroxetine 20 mg/day (Table 3).

The inclusion criteria used were: adult patients, who experience intense itching during at least one month, not associated with skin disease, able to assess the intensity, as well as having more than a month of life expectancy. Patients with known hypersensitivity to paroxetine, such as pregnant women or women who were breastfeeding, with bipolar disorder, uncontrolled epilepsy, use of MAOIs (monoamine oxidase inhibitors), chronic nausea, dependence on antihistamines and creatinine clearance estimated at less than 30 ml per minute were excluded from the study.

From a total of 26 patients included in this trial, 17 had solid tumors, 4 had hematologic malignancy and 5 did not have cancer nor had idiopathic origin for the itch.

Two primary endpoints were evaluated in this clinical trial: rating by VAS of the mean of the itch, assessed 7 days after randomization and a crossover; and individual overall response to treatment (minimum of 50% improvement in the 3 days prior compared to baseline). The study found greater effectiveness and significance of paroxetine compared with placebo (p=0.027), especially in the mean of the last 3 days of evaluation with the NAS (Numerical Analog Scale). A statistical significance was also observed (p=0.067) when a greater proportion of patients reported having preference for the week of paroxetine. Adverse events, such as drowsiness and nausea, were responsible for study discontinuation for 2 patients.^30^

## DISCUSSION

We observed in the studies compiled in this review that, recurrently, the authors stressed the importance that the itch can have in the lives of patients, regardless of the cause of the itch. They address cases that the traditional treatment with antipruritic agents does not show effectiveness in clinical practice, thus justifying the use of psychotropic drugs in their studies. Not failing to appreciate the fact that different methodologies were used in these 15 articles reviewed, we noticed that 12 of them have concluded that the use of psychotropic drugs (gabapentin in 7; pregabalin in 2 - one of them compared gabapentin and pregabalin -; doxepin in 5; paroxetine in 1; and sertraline in 1) represented a significant improvement in itch.

Even with these results, we cannot fail to emphasize the low amount of controlled clinical trials using psychotropic drugs for itch of various causes, given the importance of this theme. Furthermore, we observe the absence of clinical trials including or focusing on mental disorders that may cause or be comorbid to itch of several causes. Possibly one of the factors that justify this lack is the high prevalence of mental disorders in the general population. That said, it was observed that only one of the clinical trials used as an exclusion criterion "uncontrolled" mental illness, which may have influenced so as to cause biased results in other studies.^15^ In another study, the authors were careful to investigate the depression according to HDRS and SCID, and concluded that the underlying disease and itch could be one of the causes of the depressive symptomatology.^27^ It was observed that there is a direct and statistically significant correlation between the severity of itch and the severity of depressive symptoms.^31^

Most of patients enrolled in studies relating to itch consequent to CKD had diabetes mellitus as underlying disease of renal dysfunction. Symptoms of pain and itch resulting from peripheral neuropathy were perceived as chronic complaints and were proven through the assessment by electromyography.^15^ Solak conducted this test carefully correlating the cause of itch to medication tested for itch - gabapentin and pregabalin.^15^ It would be a great addition to the knowledge that is being established in this branch of medicine studies comparing gabapentin, pregabalin and placebo.

It is understood that the pathophysiological mechanism of itch in renal and hepatic causes is the same (neuropathic pruritus). Therefore, the use of gabapentin and pregabalin would have a good indication, although we can highlight the lack of uniformity related to difference of dose used in these trials (100 to 300 mg after hemodialysis; or daily in cases of hepatic impairment).

Gabapentin and pregabalin are part of the class of anticonvulsant, derivatives of the inhibitory neurotransmitter GABA (gamma amino butyric acid), but may have indications for peripheral neuropathy and peripheral neuralgia. Through the mechanism of action, they act so the post-synaptic excitability attenuates through depolarization changes in calcium and potassium channels and may be related to improvement of itch.^32,33^

In one of the results in that the hypotheses of the author has been refuted (that psychotropic would have greater statistical effectiveness in itch), it was discussed why the itch showed better response and behavior change after use of placebo.^27^ In this study, in a contradictory manner, even with high doses of gabapentin (maximum of 2400 mg/day), there was no significant improvement in itch. The author argues that the itch caused by the cholestatic disease is mediated by tone of opioid neuromodulators and should therefore be treated with opioid antagonists, demonstrated by controlled clinical trials. ^27,34^

To treat itch with skin disease origin (pruritoceptive), capsaicin cream, doxepin and aspirin can be used.^9^ Nevertheless, this review did not show enough clinical trials able to reinforce this behavior in clinical practice in a guided way.

Even in the treatment of itch in skin diseases, the psychotropic drug most commonly used in clinical practice is doxepin, a tricyclic antidepressant with a strong antihistamine action.^23^ Doxepin was used in two trials whose basal cause of itch was dermatologic. However, the way doxepin was used (in a study as treatment and in another as pretreatment) and the type of skin disorder diverge.^23,25^ So it is not possible to infer that doxepin shows good results related to itch. When the use of doxepin was directed to chronic urticaria, the two studies evaluated showed efficacy of antidepressant, yet both suffered from methodological flaws and follow without further replication of results.^24^ Part of this may be explained by the fact that these are two publications with about 30 years and can be used as an example of the lack of more objective tools for evaluation of itch, already present in the most recent publications.

It is important to add that psychosomatic factors modulate the perception of itch in a large amount of skin diseases.^35^ It is estimated that, in dermatological clinics, the prevalence of psychogenic itch complaints can be considered high, about 2% of patients treated. ^36^

Furthermore, the diagnosis of psychogenic itch is performed excluding other causes. However, there may occur simultaneously the other type of itch.^6,7^

Given this difficult differential diagnosis, we understand the objections to conduct controlled clinical trials for psychogenic pruritus. However, these studies are needed, as itch requires an interface between specialties as well as more evidence is needed on the effectiveness of psychotropics.

We can conclude that most of the clinical trials performed so far show statistically significant efficacy of the psychotropic in the treatment of itch for various causes. Controlled studies stood out, whose basic cause of the itch is chronic kidney disease. In these, a greater effectiveness was observed in most psychiatric drugs used compared with placebo. However, due to the small amount of controlled studies, especially in the other causes of itch, it is not possible to generalize that psychotropic drugs are effective in the treatment of itch of various causes. It was also shown that, depending on the underlying cause of itch, different medications may be used.
